# Willingness to Bear Economic Costs in the Fight Against the COVID-19 Pandemic

**DOI:** 10.3389/fpsyg.2020.588910

**Published:** 2020-10-27

**Authors:** Joanna Sokolowska, Tomasz Zaleskiewicz

**Affiliations:** ^1^Faculty of Psychology in Warsaw, Institute of Economic Psychology, SWPS University of Social Sciences and Humanities, Warsaw, Poland; ^2^Center for Research in Economic Behavior, Faculty of Psychology in Wrocław, SWPS University of Social Sciences and Humanities, Wrocław, Poland

**Keywords:** COVID-19 pandemic, tradeoffs between economic costs and health, sacred values, risk and uncertainty, compensatory and non-compensatory models of decisions

## Abstract

The COVID-19 pandemic has created a situation in which people have to choose between economic and health values. This raises the question of what psychological mechanisms determine people’s willingness to bear economic costs to protect health? To answer this question, we examined whether such willingness is better described by compensatory or lexicographic models of decision making in situations involving risk or uncertainty. We compared decisions regarding COVID-19 and occupational diseases to establish a pandemic-independent baseline and to determine whether the mechanisms behind the trade-offs are the same in both cases. Additionally, we tested whether people’s willingness to accept economic costs is related to psychological factors such as fear, feeling of control, declared knowledge about the COVID-19 pandemic, predictions concerning the expected length of the pandemic, and perceived effectiveness of actions taken to fight the coronavirus. In total, 354 Polish participants from Prolific Academic took part in this study. The results were consistent with the view that decisions are made primarily to protect sacred values and are therefore not based on compensatory models. In line with this view, participants were sensitive neither to the risk vs. uncertainty manipulation nor to the perceived effectiveness of the lockdown. Instead, their behavior was congruent with lexicographic models in which the protection of health and in particular the fight against the COVID-19 pandemic appeared to be the most important dimension, and the single criterion to be used in decision making.

## Introduction

The COVID-19 pandemic has created a situation in which people have to choose between economic and health values. For example, small business owners must decide whether to close or run their businesses, risking their own health and that of family members, employees, and customers. Our aim was to learn whether willingness to bear economic costs (unemployment and inflation) to protect health was related to probability of threat (risk vs. uncertainty) and psychological factors such as fear, feeling of control, declared knowledge about the COVID-19 pandemic, predictions concerning the expected length of the pandemic, and perceived effectiveness of actions taken to fight the coronavirus. We also examined whether such willingness was better described by compensatory or lexicographic models of decision making. We compared decisions regarding COVID-19 and occupational diseases to establish a pandemic-independent baseline and to determine whether the mechanisms behind the trade-offs are the same in both cases. Understanding the psychological mechanisms behind people’s willingness to bear economic costs is essential for developing socially acceptable policies to control the spread of COVID-19.

### Compensatory Models of Decision Making

In many situations, people have to make trade-offs. Making trade-offs between values in the same category is relatively easy, but becomes more difficult when the values belong to various categories, e.g., money vs. uncertainty or money vs. time (Luce et al., 1999, 2001). Making choices between conflicting criteria are even more difficult. This is the case for trading off costs and safety, or maximizing the returns and minimizing the risks of investments. In the medical field, conflicting criteria might be diagnostic accuracy and availability of care (Azar, 2000).

In traditional compensatory models of decision making, all criteria have to be considered when evaluating overall utility. A poor score on one criterion can be compensated by high scores on other criteria, e.g., increased economic costs are compensated by a decreased morbidity rate. Given that COVID-19 is a new disease, there is uncertainty about effective policies and preventive behaviors to limit infections, including lockdowns. Therefore, a decrease in the morbidity rate is only a probable or even an uncertain outcome, whereas the economic costs of the lockdown are a sure loss. Compensatory integration of outcomes and probabilities means that a low-utility and high-probability option may be as attractive/unattractive as a high-utility and low-probability option (for a review, see Shanteau and Pigenot, 2009). We tested this integration model in both risky and uncertain conditions. According to the compensatory approach, people should be more willing to accept economic costs in risky conditions (for a review, see Camerer and Weber, 1992).

### Lexicographic Models of Decision Making

Compensatory models of decision making raise concerns, because evaluation criteria that are hard to compare may evoke feelings of conflict (Beattie and Barlas, 2001). The most extreme examples of situations with conflicting criteria appear when people have to trade off sacred values (Tetlock, 2003) such as human life, health or freedom against secular values such as money. Weighing sacred values against other quantities is often considered either taboo (Fiske and Tetlock, 1997; Tetlock et al., 2000; Daw et al., 2015; Chorsu et al., 2018) or a repugnant transaction (Leuker et al., 2020). Tetlock et al. (2000) have proposed the sacred value protection model (SVPM), according to which “when sacred values come under secular assault, people struggle to protect their private selves and public identities from moral contamination” (Tetlock, 2003, p. 320). This is directly related to the evaluation of policies dealing with the COVID-19 pandemic. For example, many people have felt disgusted by the policies adopted in some countries to dispense with social distancing in order to reduce economic costs and advance herd immunity at the expense of putting elderly and other vulnerable people at risk of death. According to the SVPM, people should always protect health against secular values. Given that sacred values are always the same – health and human life – people’s willingness to bear economic costs to fight COVID-19 should be similar under both risk and uncertainty conditions. It should also be the same for unemployment and inflation, and for COVID-19 and occupational diseases.

Insensitivity to uncertainty is also in agreement with another lexicographic model of decision making, the priority heuristic (PH) proposed by Brandstatter et al. (2006). According to PH, people first focus on the most important aspect, which is the amount of loss. Probabilities are considered only in the next step. However, decisions made according to PH may depend on specific economic costs, because the relative importance of the unemployment, inflation and morbidity rates may differ. The relative importance of these costs and benefits may also differ between the COVID-19 and the occupational diseases conditions.

### Other Factors Related to Willingness to Accept Economic Costs to Protect Health

Following prior research on people’s reactions to the pandemic (e.g., Capraro and Barcelo, 2020a), we investigated the association between willingness to bear economic costs to protect health and various emotional and cognitive factors. One such factor is fear, the emotion that has probably been the most automatic and spontaneous response to the threat to health/life during the pandemic (Kramer et al., 2014; Taylor, 2019; Van Bavel et al., 2020). The intensity of fear is positively related to risk perception (Slovic, 1987; Marris et al., 1997; Siegrist et al., 2005). Lerner and Keltner (2000, 2001) have documented that both dispositional and incidentally evoked fear are related to higher risk estimates. People experiencing high rates of health anxiety have been found to take protective but sometimes irrational actions (Mathes et al., 2018). If fear is positively correlated with risk perception, people experiencing this emotion should be more willing to sacrifice their economic comfort and accept higher levels of unemployment and inflation to reduce health risks.

Another variable involved in the trade-offs between economic and health values is feeling of control (Slovic et al., 1985). First, one’s inability to influence the course of events is positively linked to risk perception (Slovic, 1987; Bracha and Weber, 2012; Weber, 2017). Second, lack of control is associated with low tolerance for uncertainty, which in turn correlates with excessive worry or health anxiety (Taylor, 2019). Taken together, a low level of personal control may favor one’s willingness to accept higher increases in unemployment and inflation to reduce health threats. In the present study, the role of fear and personal control was analyzed both at an abstract level (as factors linked to health irrespective of specific threats) and in direct association with COVID-19.

We also examined participants’ opinions about the effectiveness of social isolation, the expected length of the pandemic, and subjective knowledge about COVID-19. We controlled for potential relationships between willingness to trade economic and health values on the one side and demographic variables such as gender, age, personal income, place of residence and political views on the other.

According to compensatory models, these factors may influence the overall utility of the economic costs taken to reduce the morbidity rates of both COVID-19 and occupational diseases. By contrast, if decisions are made according to either SVPM or PH, they should be largely insensitive to these factors, with the exception of fear, which may reinforce the importance of health as the most important dimension in PH.

## Materials and Methods

### Participants and Design

A total of 354 Polish participants from the crowdsourcing community Prolific Academic took part in this study in exchange for £0.75 (121 women, 233 men; *M*_*age*_ = 25.02 years, SD = 9.50). Detailed information about the sample is provided in the Supplementary Material. The study had a mixed experimental design with two between-subject factors: risk/uncertainty (risk vs. uncertainty) and type of health hazard (COVID-19 vs. occupational diseases). The within-subject factor was the type of economic cost: unemployment rate or inflation rate. Following Simmons et al. (2013) recommendation, we included a minimum of 50 observations per condition. Participants were randomly assigned to one of the four conditions, which represented a combination of the two between-subject factors: COVID-19/risk (*N* = 86), COVID-19/uncertainty (*N* = 86), occupational diseases/risk (*N* = 84) and occupational diseases/uncertainty (*N* = 98). Data collection did not continue after data analysis. No data were discarded.

### Procedure

Once the participants had provided informed consent, they were presented with scenarios informing them of the economic consequences of introducing actions oriented to decreasing the morbidity rate of either COVID-19 or occupational diseases. The success of protective actions was expressed in terms of either risk or uncertainty. All participants were given two scenarios presented in a random order (see Supplementary Material). In the first scenario, a decrease in the morbidity rate came at the cost of an increase in the unemployment rate, whereas in the other scenario, the cost was an increase in the inflation rate. In both scenarios, participants were told that 100 experts were asked whether the actions taken to decrease the morbidity rate would be effective. In the risk condition, participants were informed that half of the experts predicted that the actions would yield a decrease in the morbidity rate of 30%, whereas the other half believed that the actions would not be successful. In the uncertainty condition, respondents were informed that only 20% of experts had clear opinions: 10% of them expected a 30% reduction in the morbidity rate, and 10% of them predicted no decrease in the morbidity rate. The remaining 80% of experts stated that there were no bases for making any forecasts. Thus, uncertainty was expressed as the second-order probability distribution of its possible values (e.g., Camerer and Weber, 1992). All scenarios were accompanied by graphs illustrating the frequency of expert opinions. In all graphs, the green section represented the percentage of experts who believed that the morbidity rate would decrease, the red section represented the percentage of experts who predicted no change, and the gray section (only in the uncertainty condition) represented the percentage of experts who said that making any forecast was groundless. The graphs also included numerical information about the expected change in the morbidity rate (−30% in the green area, 0% in the red area, and a question mark in the gray area). Uncertainty levels have been presented in the same form in several previous studies (e.g., Dolan and Jones, 2004; Tymula et al., 2012). The graphs can be found in the Supplementary Material.

The participants were asked to provide their opinion on the highest acceptable level of either unemployment or inflation as the cost of reducing the morbidity rate. Before providing the value, the participants were informed that in Poland, the unemployment rate in 2010–2015 ranged from 12 to 15% and was equal to 5% in 2019 and in March 2020, whereas the inflation rate ranged from −2% to 5% in 2010–2015 and was equal to 1% in 2019 and in March 2020. The participants were given the actual information in order to fix the same reference point for everybody. The unemployment and inflation rates declared by the participants as an acceptable cost of reducing the morbidity rate were dependent variables.

In the next step, the participants answered the following two questions, presented in a random order: (1) How afraid are you that you will get seriously ill and suffer serious negative consequences? (2) To what extent can you personally prevent getting seriously ill? The answers to both questions were registered on a 100-point slider scale from “very weak fear” to “very strong fear” for the first question, and from “very low impact” to “very high impact” for the second question.

The next set of five questions, presented in a random order, pertained to evaluating the consequences of COVID-19: (1) How do you evaluate the possible negative impact of the pandemic on the Polish economy? (from “very little impact” to “very high impact”); (2) In your opinion, was the social isolation policy introduced on March 11 effective? (from “completely ineffective” to “very effective”); (3) In your opinion, how long will the pandemic last? (from “very short time” to “very long time”); (4) How much fear does the COVID-19 pandemic evoke in you? (from “very little fear” to “very strong fear”); (5) How do you evaluate your knowledge about the medical consequences of COVID-19? (from “very little knowledge” to “a lot of knowledge”). Answers were provided with the aid of a 100-point slider.

The participants answered demographic questions about gender, age, socio-political opinions (using a 100-point slider from “definitely left-wing” to “definitely right-wing”), net monthly income, and place of residence. Prior research (Capraro and Barcelo, 2020b) has shown that some demographic factors (e.g., gender) may be related to willingness to take protective actions during the pandemic.

## Results

### Overview

The data were analyzed in three steps. First, willingness to bear economic costs in order to lower the morbidity rates of COVID-19 and occupational diseases was tested by comparing the rates of unemployment and inflation declared by participants as acceptable with the actual rates before the lockdown. Subsequently, using hierarchical log-linear analysis, we compared willingness to bear economic costs in the cases of COVID-19 and occupational diseases separately for unemployment and inflation. Then, a multivariate analysis of variance (MANOVA) model was used to investigate willingness to accept increases in both unemployment and inflation for the risk vs. uncertainty conditions. In the last step, we examined the relationship between willingness to bear the economic costs of the lockdown with the cognitive and affective factors, and with the socio-demographic variables. This was done with the aid of multivariate linear regression, with economic costs as dependent variables and the other factors as predictors.

### Willingness to Bear Economic Costs

**FIGURE 1:**
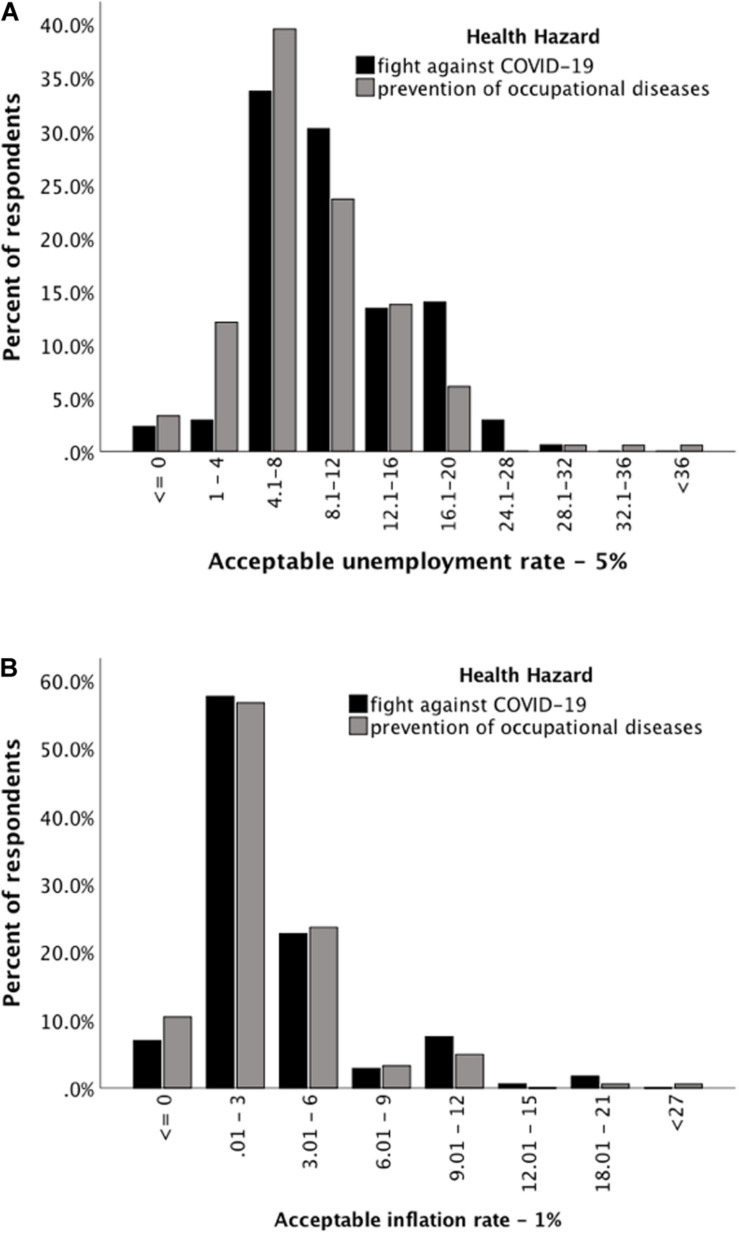
The willingness to bear economic costs higher than the status quo, i.e. unemployment rate higher than 5% and inflation rate higher than 1%.

For each participant, the actual unemployment and inflation rates in March 2020 (i.e., 5 and 1%, respectively) were subtracted from the unemployment and inflation rates declared as acceptable. Positive values of these differences meant that participants were ready to accept increases in unemployment and/or inflation. The differences are presented in Figures 1A,B.

As can be seen in Figure 1A, the majority were willing to accept an unemployment rate higher than 5% to reduce the morbidity rates of both COVID-19 (86.6%) and occupational diseases (70.9%). The participants were more frequently willing to bear a higher unemployment rate for COVID-19 than for occupational diseases (χ^2^ = 13, *df* = 1, *N* = 354, *p* < 0.001).

From Figure 1B it can be seen that the majority (70%) of participants were willing to accept a higher inflation rate to reduce the morbidity rate of both COVID-19 and occupational diseases. The frequency of responses did not differ between these two conditions (χ^2^ = 0.002, *df* = 1, *N* = 354, *p* = 0.968).

### Willingness to Bear Economic Costs Under Risk and Under Uncertainty

To examine the effect of risk/uncertainty, we performed hierarchical log-linear analysis with two factors: health hazard (COVID-19 vs. occupational diseases) and risk/uncertainty (risk vs. uncertainty) separately for willingness to accept increases in the unemployment and inflation rates. The results are summarized in Table 1.

**TABLE 1:** The three-way contingency table and results of the hierarchical loglinear analysis with three factors: health hazard (COVID-19 vs. occupational diseases), risk/uncertainty (risk vs. uncertainty), and the willingness to bear economic costs.

Health hazard	The degree of uncertainty	Willingness to bear increase in unemployment Observed frequency%		Willingness to bear increase in inflation Observed frequency%	
		No	Yes	No	Yes
COVID-19	Risk	3.7	20.6	5.9	18.4
	Uncertainty	2.8	21.5	5.4	18.9
Occupational diseases	Risk	7.3	16.4	6.5	17.2
	Uncertainty	7.6	20.1	5.4	22.3

**Hierarchical Loglinear Analysis**					

		**Willingness to bear an increase in unemployment**		**Willingness to bear an increase in inflation**	

**Term deleted from the saturated model**	**Df**	**Difference in LR χ ^2^ due to deletion of a given term:**		**Difference in LR χ ^2^ due to deletion of a given term:**	

			***p***		***p***

Hazard × Uncertainty × economic costs	1	0.062	0.804	0.399	0.528
Hazard × Uncertainty	1	0.248	0.387	0.523	0.470
Hazard × economic costs	1	13.550	>0.001	0	0.997
Uncertainty × economic costs	1	0.644	0.422	1.356	0.244

As Table 1 shows, only the interaction between the type of health hazard and the type of economic costs predicted willingness to accept a higher unemployment rate to reduce the morbidity rate: willingness was higher for COVID-19 than for occupational diseases. This interaction was insignificant for inflation. No effect of the risk/uncertainty factor was observed.

Next, MANOVA was conducted to test the model, including the type of economic cost as a within-subject factor. The limitation of comparing relative willingness to accept increases in unemployment and inflation was that each cost is measured on a different scale: unemployment ranges from 0 to 100%, whereas inflation has no specific limits. To avoid this limitation, the answers concerning inflation were rescaled. The average acceptable unemployment rate provided by the participants was 10.08, with SD = 5.81, while for the inflation rate, the average was 3.40, with SD = 3.53. Given that the ratio of these two standard deviations was 1.65, the answers concerning inflation were rescaled by this factor.

**FIGURE 2:**
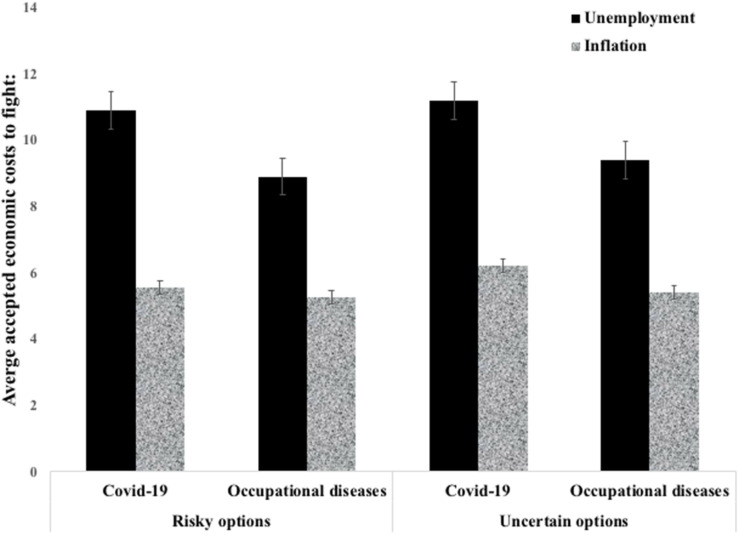
Average accepted rates of both unemployment and inflation depending on the type of disease (COVID-19 vs. occupational diseases) and the level of uncertainty (risk vs. uncertainty).

Multivariate analysis of variance with one within-subject factor (economic costs: unemployment vs. inflation) and with two between-subject factors – health hazard (COVID-19 vs. occupational diseases) and risk/uncertainty (risk vs. uncertainty) – was conducted. The average rates declared by the participants as acceptable for unemployment and inflation are shown in Figure 2.

As can be seen in Figure 2, the average acceptable rates were higher for unemployment than for inflation, for all conditions. In agreement with this observation, a significant main effect of the type of economic costs was found (*M*_(unemployment)_ = 10.08, SD = 5.81 and *M*_(inflation)_ = 5.61, SD = 5.83, *F*_(__1_,_350__)_ = 165.71, *p* < 0.001, [$\eta_{p}^{2}$] = 0.321).

We also found a significant main effect of the type of health hazard: *M*_(unemployment)_ = 11.03, SD = 5.65 and *M*_(inflation)_ = 5.88, SD = 6.00 for the COVID-19 condition; and *M*_(unemployment)_ = 9.18, SD = 5.84 and *M*_(inflation)_ = 5.36, SD = 5.66 for the occupational diseases condition, *F*_(__1_,_350__)_ = 5,49, *p* < 0.020, [$\eta_{p}^{2}$] = 0.015. Given that the differences in averages were higher for unemployment than for inflation, we also tested the interaction between the type of economic costs and the type of health hazard. This interaction was marginally significant, *F*_(__1_,_350__)_ = 3.70, *p* < 0.055, [$\eta_{p}^{2}$] = 0.010.

We found no significant main effect of risk/uncertainty for either unemployment (*M*_(risk)_ = 9.92, SD = 5.93 and *M*_(uncertainty)_ = 10.22, SD = 5.72) or inflation (*M*_(risk)_ = 5.43, SD = 6.4 and *M*_(uncertainty)_ = 5.78, SD = 5.24), *F*_(__1_,_350__)_ = 0.546, *p* = 0.461, [$\eta_{p}^{2}$] = 0.002. All interactions comprising the risk/uncertainty factor were insignificant (all *p* > 0.50), including the interaction with the type of health hazard, *F*_(__1_,_350__)_ = 0.22, *p* < 0.882, [$\eta_{p}^{2}$] = 0.000.

In summary, the participants were more willing to accept an increase in the unemployment rate than in the inflation rate. This effect was more salient for COVID-19 than for occupational diseases.

### Predictors of Willingness to Bear Economic Costs

Multivariate linear regression was used to identify predictors for the acceptable unemployment and inflation rates. These analyses were performed separately for the COVID-19 and occupational diseases conditions, because the questions directly related to the former did not apply to the latter. The responses to questions concerning fear of getting infected by COVID-19 and getting seriously ill were highly correlated (*r* = 0.60, *p* < 0.001). Therefore, the first response was used as a predictor in the regression for the COVID-19 condition, and the second in the regression for the occupational diseases condition. Risk/uncertainty, gender, and income were recoded as dummy variables. The other predictors were not transformed. First, all outliers were removed. Second, collinearity diagnostics were performed. The intercorrelations are presented in Supplementary Tables S1, S2, S5, S6 in the Supplementary Material. The coefficients, statistical significance, zero-order, partial and part correlations, and collinearity statistics are given in Tables 2, 3 only for the COVID-19 condition^1^.

**TABLE 2:** Multivariate regression: with the acceptable unemployment rate as the dependent variable for COVID-19 condition, *N* = 149.

	Unstandardized coefficients		Stand. Coef.			Correlations			Collinearity statistics		Eig. value	Con. Index
	*B*	Std. Error	β	*T*	*p*	Zero-order	Partial	Part	Tolerance	VIF		
Constant	9.493	3.823		2.48	0.014				0.923	1.08	10.02	1.00
Risk/Uncertainty	–0.192	0.884	–0.019	–0.22	0.829	–0.052	–0.019	–0.018	0.886	1.13	0.55	4.3
Gender	1.476	1.025	0.126	1.44	0.152	0.122	0.122	0.119	0.865	1.16	0.437	4.8
Income	0.559	0.917	0.054	0.61	0.544	0.043	0.052	0.050	0.766	1.31	0.304	5.8
Fear: COVID-19	–0.001	0.021	–0.002	–0.02	0.981	0.043	–0.002	–0.002	0.783	1.28	0.141	8.4
Effectiveness of the lockdown	0.034	0.021	0.152	1.63	0.105	0.173	0.138	0.134	0.627	1.60	0.186	7.3
Predicted length of pandemic	0.016	0.028	0.060	0.57	0.568	0.071	0.049	0.047	0.847	1.18	0.088	10.7
Impact on economy	–0.039	0.027	–0.127	–1.41	0.160	–0.135	–0.120	–0.117	0.927	1.08	0.061	12.8
Knowledge about pandemic	–0.008	0.022	–0.031	–0.37	0.716	–0.048	–0.031	–0.030	0.817	1.22	0.120	9.2
Age	0.032	0.063	0.046	0.51	0.612	–0.010	0.043	0.042	0.887	1.13	0.055	13.5
Political views	–0.001	0.019	–0.003	–0.03	0.977	0.011	–0.002	–0.002	0.922	1.08	0.033	17.5
Control over getting sick	–0.011	0.023	–0.041	–0.48	0.632	–0.029	–0.041	–0.040			0.010	31.0

*Dummy coded: Hazard (1 = COVID-19); Risk/Uncertainty (1 = Risk); Income (1 = below average); Gender (1 = male).*

**TABLE 3:** Multivariate regression with the acceptable inflation rate as the dependent variable for COVID-19 condition, *N* = 137.

	Unstandardized coefficients		Stand. Coef.			Correlations			Collinearity statistics			
	*B*	Std. Error	β	*T*	*p*	Zero-order	Partial	Part	Tolerance	VIF	Eig. value	Con. Index
Constant	5.086	2.087		2.44	0.016						10.03	1.0
Risk/Uncertainty	–0.054	0.467	–0.010	–0.116	0.908	–0.066	–0.010	–0.010	0.930	1.08	0.538	4.3
Income	0.457	0.486	0.084	0.942	0.348	0.045	0.084	0.078	0.863	1.16	0.459	4.7
Gender	1.478	0.556	0.235	2.66	0.009	0.247	0.231	0.220	0.881	1.14	0.290	5.9
Fear: COVID-19	–0.005	0.011	–0.044	–0.464	0.643	–0.024	–0.041	–0.038	0.765	1.31	0.177	7.5
Effectiveness of the lockdown	0.003	0.011	0.021	0.222	0.825	0.063	0.020	0.018	0.738	1.36	0.135	8.6
Predicted length of pandemic	0.017	0.014	0.126	1.20	0.234	0.053	0.106	0.099	0.622	1.61	0.121	9.1
Impact on economy	–0.025	0.012	–0.186	–2.14	0.034	–0.157	–0.161	–0.151	0.912	1.10	0.065	12.4
Knowledge about pandemic	–0.027	0.015	–0.165	–1.83	0.070	–0.203	–0.188	–0.178	0.842	1.19	0.085	10.9
Age	0.020	0.036	0.051	0.544	0.588	–0.027	0.049	0.045	0.792	1.26	0.052	13.4
Political views	–0.001	0.011	–0.008	–0.093	0.926	0.026	–0.008	–0.008	0.905	1.11	0.034	17.3
Control over getting sick	–0.010	0.012	–0.070	–0.805	0.422	–0.104	–0.072	–0.067	0.909	1.10	0.010	32.1

*Dummy coded: Hazard (1 = COVID-19); Risk/Uncertainty (1 = Risk); Income (1 = below average); Gender (1 = male).*

As can be seen in Tables 2, 3, for both unemployment and inflation most variables passed the collinearity test, the exceptions being personal control over getting seriously ill, and political views.

Despite the significant correlations (see Supplementary Table S1) between the acceptable unemployment rate on the one side and the perceived effectiveness of the lockdown (*r* = 0.17, *p* < 0.05, *N* = 149) and the perceived impact on the economy on the other (*r* = −0.14, *p* < 0.05, *N* = 149), no significant model was found for unemployment (adj*R*^2^ = −0.006, *F*_(__11_,_237__)_ = 0.92, *p* = 0.527). This could be explained by the fact that the partial and part correlations were lower than the zero-order correlations (see Table 2)^2^.

For inflation, a marginally significant model was found (adj*R*^2^ = 0.065, *F*_(__11_,_125__)_ = 1.86 *p* = 0.051). Although the model fit was significant, most of the coefficients for the predictors were non-significant (see Table 3). The two exceptions were gender, as men accepted a higher inflation rate than women (*B* = 1.478, *p* = 0.009 with lower and upper confidence intervals 0.378 and 2.578)^3^, and perceived impact on the economy (β = −0.186. *p* = 0.034). For the second predictor, the partial and part correlations were lower than the zero-order correlation, pointing at an input of other variables in this correlation (Table 3). Indeed, as can be seen in Supplementary Table S2, the perceived impact on the economy was positively correlated with the perceived length of the pandemic (*r* = 0.27, *p* < 0.01, *N* = 137), and negatively correlated with the perceived effectiveness of the lockdown (*r* = −0.18, *p* < 0.05, *N* = 137). The perceived length of the pandemic and the perceived effectiveness of the lockdown were significantly correlated (*r* = 0.26, *p* < 0.01, *N* = 137). Such relationships suggest that the perceived impact on the economy had an indirect effect on DV. To test this explanation, Model 2 from Hayes Process V. 3.5 (Hayes, 2018) was applied, with acceptable inflation rate as DV, perceived impact on the economy as IV, and both perceived length of the pandemic and perceived effectiveness of the lockdown as moderators. In this model, neither the direct effect of the perceived impact on the economy (*B* = 0.067, SE = 0.046, *p* = 0.147, with lower and upper bounds of 95% CI: −0.02 and 0.16, respectively) nor its interaction with the perceived effectiveness of the lockdown (*B* = −0.001, SE = 0.001, *p* = 0.192, with lower and upper bounds of 95% CI: −0.002 and 0.001, respectively) were significant. Only the effect of the perceived length of the pandemic (*B* = 0.11, SE = 0.05, *p* = 0.04, with lower and upper bounds of 95% CI: 0.01 and 0.20, respectively) and its interaction with the perceived impact on the economy (*B* = −0.001, SE = 0.001, *p* = 0.04, with lower and upper bounds of 95% CI: −0.0025 and −0.0001, respectively) were significant. Therefore, there was no direct effect of the perceived impact on the economy on the acceptable inflation rate.

For the occupational diseases condition, no significant model was found for DVs (adj*R*^2^ = 0.019, *F*_(__7_,_144__)_ = 1.42, *p* = 0.200 for unemployment, and adj*R*^2^ = −0.007, *F*_(__11_,_237__)_ = 1.14, *p* = 0.340 for inflation). See Supplementary Tables S3, S4 for details.

### Summary of the Results and Discussion

The results of this research revealed that the majority of participants were willing to accept economic costs to fight COVID-19 and to prevent occupational diseases. At the same time, the responses were not sensitive to the risk/uncertainty factor and were not correlated with economic factors, specifically the lockdown’s impacts on the economy and people’s incomes.

These results are inconsistent with compensatory decision models. Instead, they agree with lexicographic models, such as SVPM and PH. In line with this view, the participants were sensitive neither to the risk vs. uncertainty manipulation nor to the perceived effectiveness of the lockdown, which may moderate a subjective evaluation of the probability that economic sacrifices will reduce health risks. According to the SVPM, the protection of sacred values is not a function of payoffs weighted by their probabilities. Consequently, factors such as knowledge about the pandemic and perceived control over being infected by COVID-19 do not appear to shape people’s decisions. Rather, people focus on protecting their health, irrespective of how fearful they are, how much personal control they have, or how they evaluate the context of the pandemic. In PH, the protection of health and in particular fighting against COVID-19 may be the most important dimensions used in the first step of a lexicographic strategy. The decision is made in the first step if the difference in the most important dimension is sufficiently salient (Brandstatter et al., 2006). The greater willingness to accept a higher unemployment rate to fight COVID-19 than to prevent occupational diseases observed may indicate the higher relative importance of health in the former case. In summary, our findings seem to be in accordance with lexicographic models of decision making.

The lack of effect of the risk/uncertainty factor can also be explained in an alternative way. We cannot exclude that this finding reflects participants’ lack of trust in experts’ opinions. Prior research suggests that laypeople have limited trust in expert opinions, and that advice taking is sensitive to consistency between these opinions and people’s own beliefs (Zaleskiewicz and Gasiorowska, 2018, 2020).

One factor examined in this study, namely fear, requires additional consideration. According to Kahneman and Frederick (2002) model of heuristic judgment, relevant but hard-to-process attributes are substituted by irrelevant ones that can be easily processed. Affect is a natural dimension frequently used by decision makers as a substitute. Therefore, fear is a good candidate to guide hard choices either through the affect heuristic (Alhakami and Slovic, 1994) or as the most important dimension in a lexicographic model. In line with the affect heuristic, people base their judgments of an activity on how they feel about it; affect then guides their perceptions of benefits and risks. Therefore, fear may bias judgments of payoffs and probabilities in favor of willingness to bear economic costs. Fear may also be the single criterion for decision making used in a lexicographic model.

In contrast to these expectations, we identified no impact of fear. One possible explanation is that the level of fear was relatively low (*M* = 41.95 on a scale from 0 to 100), possibly owing to the youngness of the participants: 52% of them were 23 years old or younger. Additionally, the study was carried out in late May 2020, when the number of daily new cases stabilized, and the numbers of active cases and deaths in Poland were low in comparison to many other European countries. As a result, fear was not related to willingness to make sacrifices.

Our findings concerning willingness to bear economic costs appeared to be in disagreement with increasing social protests against the lockdown. To understand this, more research is necessary, involving respondents belonging to different age groups and social strata, and with different health conditions. It would also be helpful to treat the situation as a dynamic one, and therefore conduct longitudinal studies.

Similarly to earlier research in the field, we also found associations between reactions during the pandemic and some socio-demographic variables (see Capraro and Barcelo, 2020a,b). For example, women were less willing than men to accept economic costs in order to lower the COVID-19 morbidity rate, but declared higher levels of fear. The latter effect may be a good predictor of gender differences in obeying protective recommendations during the pandemic.

## Data Availability Statement

The raw data supporting the conclusions of this article will be made available by the authors, without undue reservation.

## Ethics Statement

The studies involving human participants were reviewed and approved by Research Ethics Review Board of SWPS University of Social Sciences and Humanities, Wrocław, Poland. The patients/participants provided their written informed consent to participate in this study.

## Author Contributions

All authors listed have made a substantial, direct and intellectual contribution to the work, and approved it for publication.

## Conflict of Interest

The authors declare that the research was conducted in the absence of any commercial or financial relationships that could be construed as a potential conflict of interest.

## Footnotes

1 The results for the occupational diseases condition are presented in Supplementary Tables S3–S6 in the Supplementary Material.

2 Even though gender was not a significant predictor in the models found for both unemployment and inflation, for the COVID-19 condition, significant gender differences in willingness to bear economic costs were found. Indeed, 74.4% of women and 89.2% of men were willing to accept a higher unemployment rate (χ^2^ = 5.07, *df* = 1, *p* = 0.024, *N* = 150).

3 A total of 64.1% of women and 80.2% of men were willing to accept a higher inflation rate (χ^2^ = 4.09, *df* = 1, *p* = 0.043, *N* = 150).
