# Supplementary material for: Willingness to Bear Economic Costs in the Fight Against the COVID-19 Pandemic
Source: Front Psychol. 2020 Oct 27;11:588910. doi: 10.3389/fpsyg.2020.588910 (PMC7653024; doi:10.3389/fpsyg.2020.588910)
Supplement: Supplementary file 1 [file Data_Sheet_1.docx]

**Supplementary Materials**

**Materials used in the experiment**

***Scenarios and questions to scenarios***

1. ***Condition: Risk x COVID-19; unemployment***

One of the potential consequences of fighting against COVID-19, with the goal to reduce morbidity, is an increase in unemployment as a result of employees staying at home. One hundred experts were asked whether encouraging employees to stay at home would be effective in fighting the virus. Half of the experts (i.e., 50%) believed it would be effective and predicted that the morbidity rate will decrease by 30%. The rest of the experts (i.e., 50%) believed it would be not effective at all. The distribution of experts’ opinions is illustrated in the graph below.

In your opinion, what is the highest acceptable unemployment rate as a result of the decrease in morbidity? Enter the percentage value in the field below.

(In years 2010–2015, unemployment in Poland ranged from 12% to 15%. In 2019, it was equal to 5%).

1. ***Condition: Risk x COVID-19; inflation***

One of the potential consequences of fighting against COVID-19, with the goal to reduce morbidity, is an increase in prices (i.e., increased inflation) as a result of limiting the import of cheap goods from Asian countries. One hundred experts were asked whether such a strategy would be effective in fighting the virus. Half of the experts (i.e., 50%) believed it would be effective and predicted that the morbidity rate will decrease by 30%. The rest of the experts (i.e., 50%) believed it would be not effective at all. The distribution of experts’ opinions is illustrated in the graph below.

In your opinion, what is the highest acceptable inflation rate as a result of limiting the import of cheap goods from Asian countries? Enter the percentage value in the field below.

(In years 2010–2015, inflation in Poland ranged from -2% to 5%. In 2019, it was equal to 1%).

1. ***Condition: Uncertainty x COVID-19; unemployment***

One of the potential consequences of fighting against COVID-19, with the goal to reduce morbidity, is an increase in unemployment as a result of employees staying at home. One hundred experts were asked whether encouraging employees to stay at home would be effective in fighting the virus. Ten percent of the experts believed it would be effective and predicted that the morbidity rate will decrease by 30%. Another 10% believed it would be not effective at all. The rest of the experts concluded that there is no basis for making forecasts. The distribution of experts’ opinions is illustrated in the graph below.

In your opinion, what is the highest acceptable unemployment rate as a result of the decrease in morbidity? Enter the percentage value in the field below.

(In years 2010–2015, unemployment in Poland ranged from 12% to 15%. In 2019, it was equal to 5%).

1. ***Condition: Uncertainty x COVID-19; inflation***

One of the potential consequences of fighting against COVID-19, with the goal to reduce morbidity, is an increase in prices (i.e., increased inflation) as a result of limiting the import of cheap goods from Asian countries. One hundred experts were asked whether such a strategy would be effective in fighting the virus. Ten percent of the experts believed it would be effective and predicted that the morbidity rate will decrease by 30%. Another 10% believed it would be not effective at all. The rest of the experts concluded that there is no basis for making forecasts.

In your opinion, what is the highest acceptable inflation rate as a result of limiting the import of cheap goods from Asian countries? Enter the percentage value in the field below.

(In years 2010–2015, inflation in Poland ranged from -2% to 5%. In 2019, it was equal to 1%).

1. ***Condition: Risk x occupational diseases; unemployment***

One of the potential consequences of fighting against occupational diseases, with the goal to reduce morbidity, is an increase in unemployment as a result of the closure of companies where the work is harmful to health. One hundred experts were asked whether the closure of such companies would be effective in fighting occupational diseases. Half of the experts (i.e., 50%) believed it would be effective and predicted that the morbidity rate will decrease by 30%. The rest of the experts (i.e., 50%) believed it would be not effective at all. The distribution of experts’ opinions is illustrated in the graph below.

In your opinion, what is the highest acceptable unemployment rate as a result of the decrease in morbidity? Enter the percentage value in the field below.

(In years 2010–2015, unemployment in Poland ranged from 12% to 15%. In 2019, it was equal to 5%).

1. ***Condition: Risk x occupational diseases; inflation***

One of the potential consequences of fighting against occupational diseases, with the goal to reduce morbidity, is an increase in prices (i.e., increased inflation) as a result of statutory increases in the prices of products, the production of which is harmful to employees. One hundred experts were asked whether such an increase in prices would be effective in fighting occupational diseases. Half of the experts (i.e., 50%) believed it would be effective and predicted that the morbidity rate will decrease by 30%. The rest of the experts (i.e., 50%) believed it would be not effective at all. The distribution of experts’ opinions is illustrated in the graph below.

In your opinion, what is the highest acceptable inflation rate as a result of the increase in the prices of products, the production of which is harmful to employees? Enter the percentage value in the field below.

(In years 2010–2015, inflation in Poland ranged from -2% to 5%. In 2019, it was equal to 1%).

1. ***Condition: Uncertainty x occupational diseases; unemployment***

One of the potential consequences of fighting against occupational diseases, with the goal to reduce morbidity, is an increase in unemployment as a result of the closure of companies where work is the most harmful to health. One hundred experts were asked whether the closure of such companies would be effective in fighting occupational diseases. Ten percent of the experts believed it would be effective and predicted that the morbidity rate will decrease by 30%. Another 10% believed it would be not effective at all. The rest of the experts concluded that there is no basis for making forecasts.

In your opinion, what is the highest acceptable unemployment rate as a result of the decrease in morbidity? Enter the percentage value in the field below.

(In years 2010–2015, unemployment in Poland ranged from 12% to 15%. In 2019, it was equal to 5%).

1. ***Condition: Uncertainty x occupational diseases; inflation***

One of the potential consequences of fighting against occupational diseases, with the goal to reduce morbidity, is an increase in prices (i.e. increased inflation) as a result of statutory increases in the prices of products, the production of which is harmful to employees. One hundred experts were asked whether such an increase in prices would be effective in fighting occupational diseases. Ten percent of the experts believed it would be effective and predicted that the morbidity rate will decrease by 30%. Another 10% of the experts believed it would be not effective at all. The rest of the experts concluded that there is no basis for making forecasts.

In your opinion, what is the highest acceptable inflation rate as a result of the increase in the prices of products, the production of which is harmful to employees? Enter the percentage value in the field below.

(In years 2010–2015, inflation in Poland ranged from -2% to 5%. In 2019, it was equal to 1%).

***Graphs illustrating the distribution of experts’ opinions presented in the experimental scenarios***

1. ***The risk condition***

***
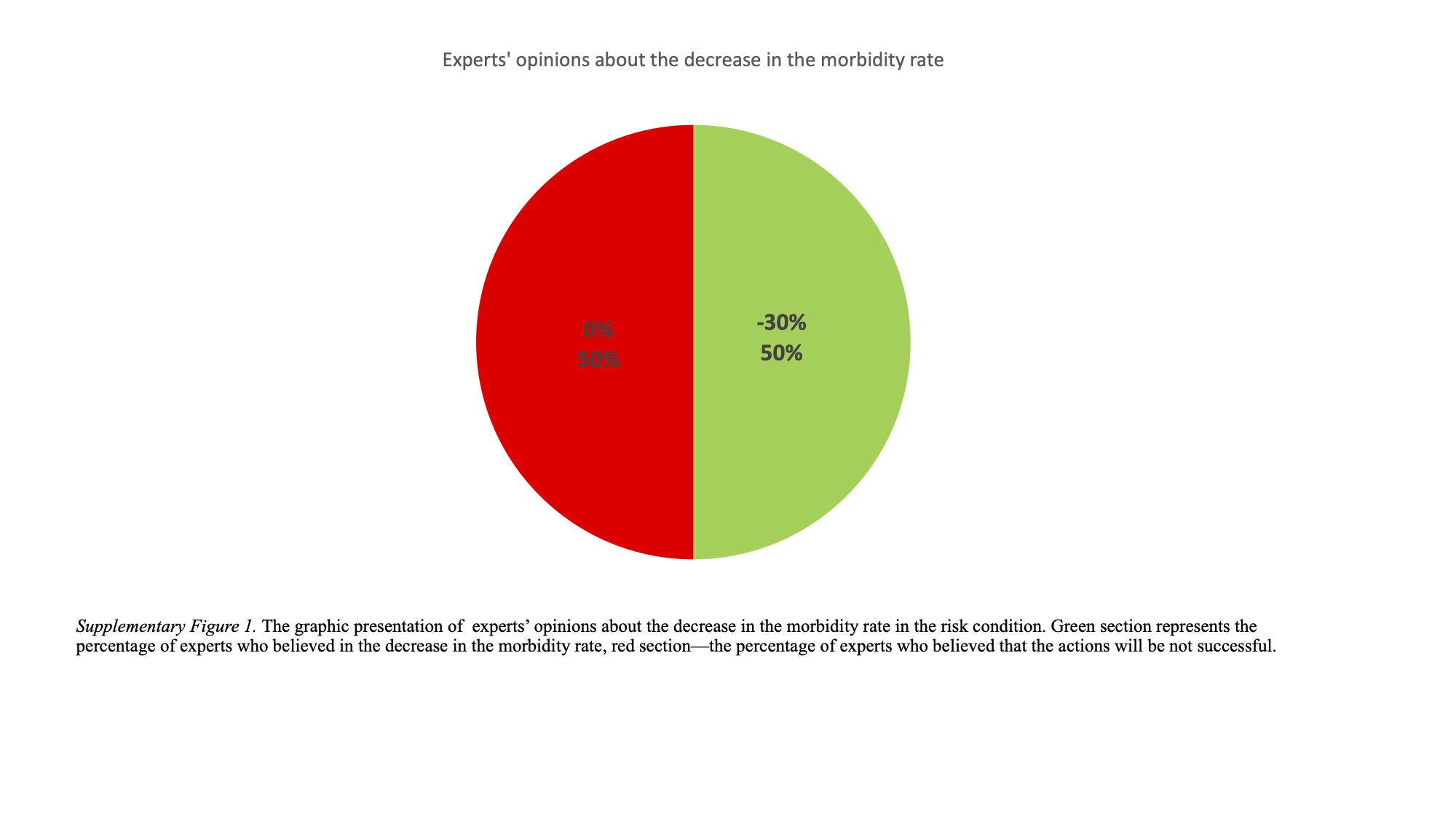
***

1. ***The uncertainty condition***


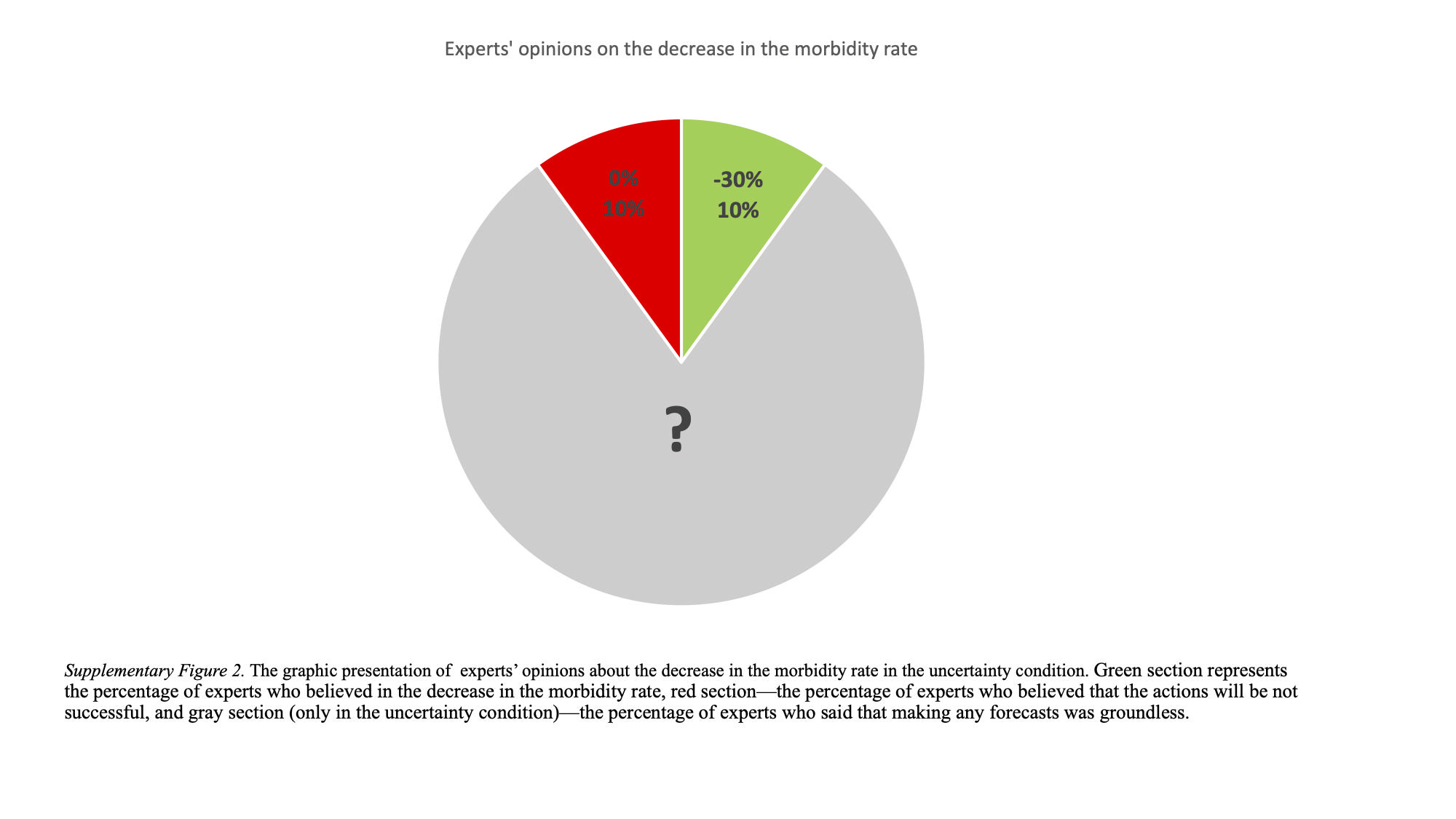


***Questions concerning fear and feeling of control (in a random order)***

(1) How afraid are you that you will get seriously ill and suffer serious negative consequences? (responses ranging from 0—“very weak fear” to 100—“very strong fear”)

(2) To what extent can you personally prevent getting seriously ill? (responses ranging from 0—“very low impact” to 100—“very high impact”)

***Additional questions (in a random order)***

(1) How do you evaluate the possible negative impact of the pandemic on the Polish economy? (responses ranging from 0—“very little impact” to 100—“very high impact”)

(2) In your opinion, was the social isolation policy introduced on March 11 effective? (responses ranging from 0—“completely ineffective” to 100—“very effective”)

(3) In your opinion, how long will the pandemic last? (responses ranging from 0—“very short time” to 100—“very long time”)

(4) How much fear does the COVID-19 pandemic evoke in you? (responses ranging from 0—“very little fear” to 100—“very strong fear”)

(5) How do you evaluate your knowledge about the medical consequences of COVID-19? (responses ranging from 0—“very little knowledge” to 100—“a lot of knowledge”)

***Demographic questions***

(1) Gender: female or male

(2) Age in years

(3) Evaluate your socio-political views (responses ranging from 0—“definitely left-wing” to 100—“definitely right-wing”)

(4) Provide your monthly net income ($1 = approximately 3.70 PLN)

- less than 1,000 PLN

- between 1,000 PLN and 2,000 PLN

- between 2,000 PLN and 3,000 PLN

- between 3,000 PLN and 4,000 PLN

- between 4,000 PLN and 5,000 PLN

- between 5,000 PLN and 6,000 PLN

- more than 6,000 PLN

(5) Provide your current place of residence

- town with up to 1,000 inhabitants

- town with 1,000 to 10,000 inhabitants

- town with 10,000 to 50,000 inhabitants

- town with 50,000 to 100,000 inhabitants

- town with more than 100,000 inhabitants

**Sociodemographic characteristic of the sample**

**Gender and age**

A total of 121 women and 233 men took part in this experiment. Five women did not give their age. The average age of the participants was 25 years, *SD*=9.5; 52% of the respondents were younger than 23 years (*SK*=3.91). The women were older than the men on average (*M*=25.96 and 24.24, *SD*=7.95 and 7.01, respectively; *t*=1.97, *df*=347, *p*=.050).

**Place of residence**

Most respondents lived in cities with more than 100,000 inhabitants (47.4%), 11.1% lived in cities with population between 50,000 and 100,000 inhabitants, 15.6% lived in towns with population between 10,000 and 50,000 inhabitants, 13.4% lived in small towns with less than 10,000 inhabitants, and 12.5% lived in villages.

**Income**

Fifty respondents did not answer the question about income. The majority of those who answered the question declared an income below the national average of 1,000 euros (59%), 19% declared an income equal to the average, and 22% declared an income above the national average. The distribution of income for women and men is presented in Supplementary Figure 3.


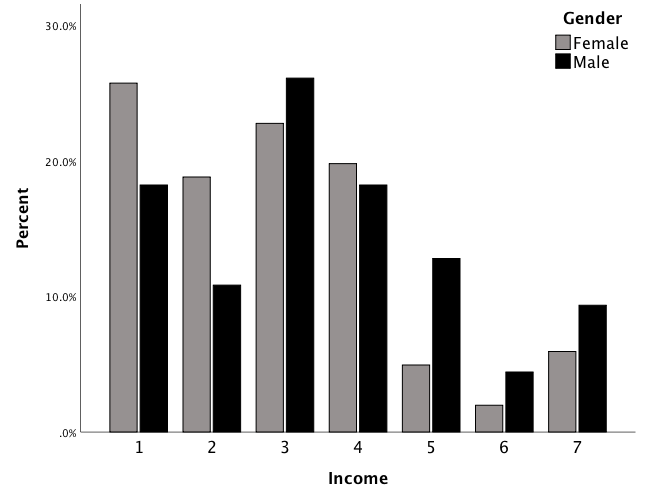


*Supplementary Figure 3.* Distribution of income among women and men.

Women declared a lower income than men on average (*M*=2.89 and 3.47, *SD*=1.66 and 1.76, respectively; *t*=-2.8, *df*=302, *p*=.006).

**Political views**

On the response scale from 0 (definitely left-wing) to 100 (definitely right-wing), 24% of the respondents chose the value 50. Of the others, 43% chose values lower than 50 and 33% chose values higher than 50. The distribution of answers was very wide and the shape differed from the Gaussian one. This distribution is given in Supplementary Figure 4.


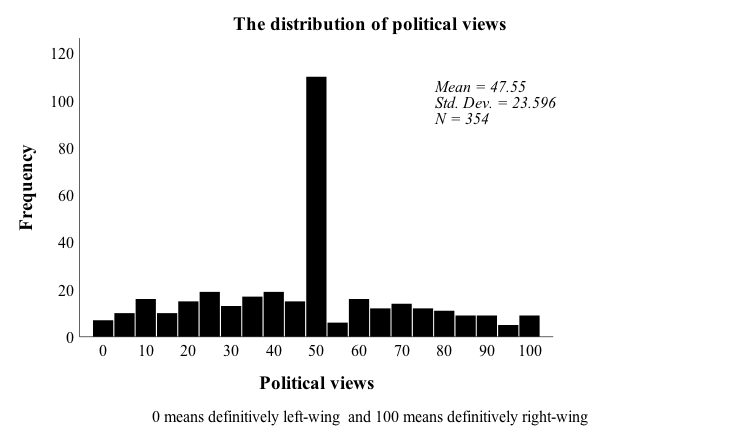


*Supplementary Figure 4.* Distribution of political views.

Women held more leftist political views than men on average (*M*=38.75 and 52.11, *SD*=20.29 and 23.94, respectively; *t*=-5.52, *df*=352, *p*<.001).

**Fear of getting seriously ill**

The distribution of respondents’ responses is given in Supplementary Figure 5.


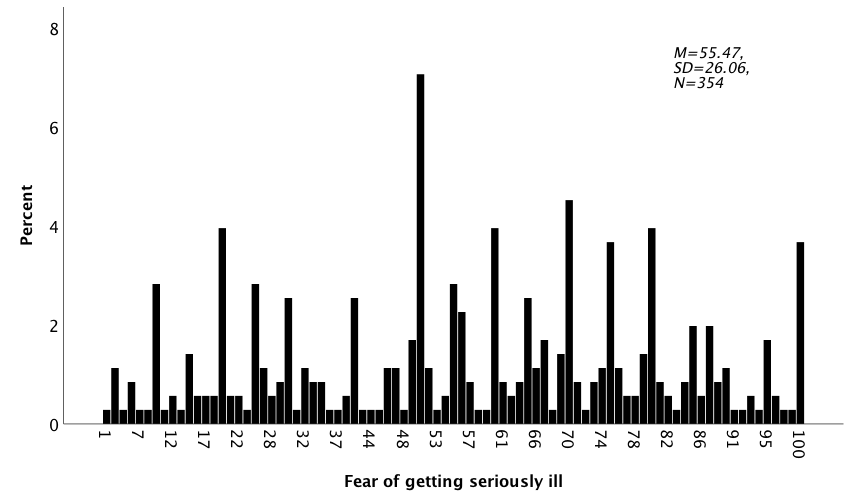


*Supplementary Figure 5.* Distribution of responses about getting seriously ill.

Women declared a significantly higher level of fear than men on average (*M*=64.83 and 50.61, *SD*=22.39 and 26.55, respectively; *t*=5.31, *df*=352, *p*<.001).

**Fear of getting infected by COVID-19**

The distribution of respondents’ responses is given in Supplementary Figure 6.


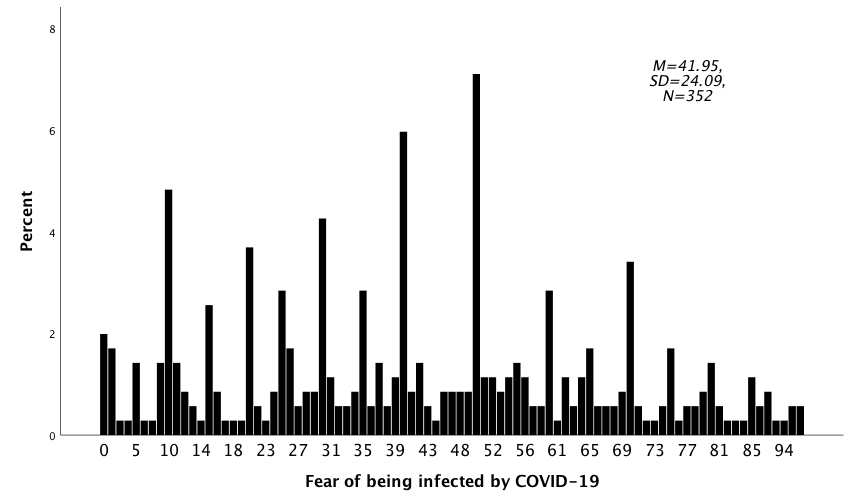


*Supplementary Figure 6.* Distribution of responses about getting infected by COVID-19.

Women declared a significantly higher level of fear than men on average (*M*=48.65 and 38.53, *SD*=23.99 and 23.46, respectively; *t*=3.77, *df*=350, *p*<.001).

Table S1. *Pearson r* correlations for the acceptable unemployment rate and other variables, the COVID 19 condition (*N*=149)

|  | 1 | 2 | 3 | 4 | 5 | 6 | 7 | 8 | 9 | 10 | 11 |
| --- | --- | --- | --- | --- | --- | --- | --- | --- | --- | --- | --- |
| 1. Unemployment |  |  |  |  |  |  |  |  |  |  |  |
| 2. Risk/uncertainty | -.05 |  |  |  |  |  |  |  |  |  |  |
| 3. Gender | .12 | -.01 |  |  |  |  |  |  |  |  |  |
| 4. Income | .04 | -.12 | -.11 |  |  |  |  |  |  |  |  |
| 5. Lockdown effectiveness | **.17*** | -.09 | -.05 | .13 |  |  |  |  |  |  |  |
| 6. Fear of getting COVID 19 | .04 | -.09 | -.07 | -.00 | **.19*** |  |  |  |  |  |  |
| 7. Knowledge about pandemic | -.05 | .03 | -.03 | -.05 | -.09 | .11 |  |  |  |  |  |
| 8. Length of pandemic | .07 | **-.22**** | .02 | -.04 | **.23**** | **.46**** | .10 |  |  |  |  |
| 9. Impact on economy | **-.14*** | -.02 | -.07 | .03 | **-.15*** | .07 | .04 | **.27**** |  |  |  |
| 10. Age | -.01 | -.03 | -.06 | **-.28**** | **-.19*** | .12 | **.17*** | .**18*** | .117 |  |  |
| 11. Political views | .01 | -.03 | **.28**** | -.11 | -.01 | -.06 | .02 | -.08 | .06 | -.01 |  |
| 12. Control over getting sick | -.03 | .00 | -.04 | .02 | **.19*** | -.06 | .10 | -.049 | .00 | -.11 | .02 |

Note. *p ≤ .05; **p < .01. Dummy variables coded: Risk/Uncertainty (1=Risk); Gender (1 = male); Income (1=below average)

Table S2. *Pearson r* correlations for the acceptable inflation rate and other variables, the COVID 19 condition (*N*=137)

|  | 1 | 2 | 3 | 4 | 5 | 6 | 7 | 8 | 9 | 10 | 11 |
| --- | --- | --- | --- | --- | --- | --- | --- | --- | --- | --- | --- |
| 1. Inflation |  |  |  |  |  |  |  |  |  |  |  |
| 2. Risk/uncertainty | -.066 |  |  |  |  |  |  |  |  |  |  |
| 3. Income | .045 | -.082 |  |  |  |  |  |  |  |  |  |
| 4. Gender | **.247**** | -.055 | -.106 |  |  |  |  |  |  |  |  |
| 5. Fear of getting COVID 19 | -.024 | -.119 | .020 | -.069 |  |  |  |  |  |  |  |
| 6. Lockdown effectiveness | .063 | -.108 | .130 | -.043 | **.196*** |  |  |  |  |  |  |
| 7. Length of pandemic | .053 | **-.208**** | -.034 | .011 | **.462**** | **.263**** |  |  |  |  |  |
| 8. Knowledge about pandemic | **-.203**** | .066 | -.043 | -.058 | .110 | -.058 | **.151*** |  |  |  |  |
| 9. Impact on economy | **-.157*** | -.022 | .027 | -.058 | .111 | **-.178*** | **.267**** | .068 |  |  |  |
| 10. Age | -.027 | -.035 | **-.305**** | -.096 | .120 | **-.217*** | **.156*** | **.159*** | .108 |  |  |
| 11. Political views | 0.26 | .004 | -111 | **.261**** | -.027 | -.008 | -.067 | .022 | .043 | .023 |  |
| 12. Control over getting sick | -.104 | .037 | .026 | -.050 | -.053 | **.202*** | -.057 | .124 | -.032 | -.117 | .009 |

Note. *p ≤ .05; **p < .01. Dummy variables coded: Risk/Uncertainty (1=Risk); Gender (1 = male); Income (1=below average)

Table S3. Multivariate Regression with the declared acceptable unemployment rate as the dependent variable: the unstandardized and standardized coefficients, significance, zero-order, partial and part correlations, and collinearity statistics, the occupational diseases condition *(N=*152)

|  | Unstandardized Coefficients | | Stand. Coef. |  |  | Correlations | | | Collinearity Statistics | | | |
| --- | --- | --- | --- | --- | --- | --- | --- | --- | --- | --- | --- | --- |
|  | *B* | *Std. Error* | *Beta* | *t* | *Sig.* | *Zero-order* | *Partial* | *Part* | *Tole-rance* | *VIF* | *Eigen-value* | *Condition Index* |
| Constant | 7.980 | 2.442 |  |  |  |  |  |  |  |  | 6.29 | 1.00 |
| Risk/uncertainty | -.940 | .776 | -.100 | -1.21 | .228 | -.114 | -.100 | -.098 | .954 | 1.048 | .508 | 3.52 |
| Gender | -.215 | .849 | -.022 | -.25 | .801 | .008 | -.021 | -.020 | .827 | 1.209 | .429 | 3.83 |
| Income | -1.525 | .852 | -.156 | -1.79 | .075 | -.136 | -.148 | -.144 | .857 | 1.167 | .348 | 4.25 |
| Fear about getting seriously sick | .015 | .015 | .080 | .94 | .347 | .111 | .078 | .076 | .895 | 1.118 | .162 | 6.23 |
| Personal control over getting sick | .021 | .020 | .087 | 1.06 | .293 | .091 | .088 | .085 | .966 | 1.035 | .151 | 6.45 |
| Age | -.044 | .034 | -.112 | -1.30 | .197 | -.065 | -.107 | -.104 | .876 | 1.142 | .092 | 8.26 |
| Political views | .024 | .018 | .113 | 1.34 | .181 | .072 | .111 | .108 | .914 | 1.094 | .019 | 18.07 |

Note. Dummy variables coded: Risk/Uncertainty (1=Risk); Gender (1 = male); Income (1=below average)

Table S4. Multivariate Regression with the declared acceptable inflation rate as the dependent variable: the unstandardized and standardized coefficients, significance, zero-order, partial and part correlations, and collinearity statistics, the occupational diseases condition *(N=*152)

|  | Unstandardized Coefficients | | Stand. Coef. |  |  | Correlations | | | Collinearity Statistics | | | |
| --- | --- | --- | --- | --- | --- | --- | --- | --- | --- | --- | --- | --- |
|  | *B* | *Std. Error* | *Beta* | *t* | *Sig.* | *Zero-order* | *Partial* | *Part* | *Tole-rance* | *VIF* | *Eigen-value* | *Condition Index* |
| Constant | 2.368 | 1.254 |  | 1.89 | .061 |  |  |  |  |  | 6.28 | 1.00 |
| Risk/uncertainty | -.589 | .400 | -.122 | -1.47 | .143 | -.123 | -.122 | -.119 | .953 | 1.05 | .510 | 3.51 |
| Gender | -.061 | .439 | -.012 | -.14 | .890 | .017 | -.012 | -.011 | .823 | 1.21 | .427 | 3.84 |
| Income | -.505 | .436 | -.101 | -1.16 | .248 | -.086 | -.096 | -.094 | .859 | 1.16 | .351 | 4.23 |
| Fear about getting seriously sick | .005 | .008 | .060 | .69 | .489 | .079 | .058 | .056 | .894 | 1.12 | .162 | 6.22 |
| Personal control over getting sick | .008 | .010 | .065 | .79 | .434 | .065 | .065 | .064 | .966 | 1.04 | .153 | 6.41 |
| Age | -.016 | .018 | -.079 | -.91 | .364 | -.046 | -.076 | -.074 | .877 | 1.140 | .094 | 8.18 |
| Political views | .016 | .009 | .148 | 1.74 | .084 | .111 | .144 | .141 | .913 | 1.10 | .019 | 18.04 |

Note. Dummy variables coded: Risk/Uncertainty (1=Risk); Gender (1 = male); Income (1=below average)

Table S5. *Pearson r* correlations for the acceptable unemployment rate and other variables, the occupational diseases condition (*N*=152)

|  | 1 | 2 | 3 | 4 | 5 | 6 | 7 |
| --- | --- | --- | --- | --- | --- | --- | --- |
| 1. Unemployment |  |  |  |  |  |  |  |
| 2. Risk/uncertainty | -.114 |  |  |  |  |  |  |
| 3. Gender | .008 | .062 |  |  |  |  |  |
| 4. Income | **-.136*** | **.148*** | -.086 |  |  |  |  |
| 5. Fear about getting seriously sick | .111 | -.105 | **-.257**** | **-.154*** |  |  |  |
| 6. Personal control over getting sick | .091 | -.057 | -.009 | .055 | .019 |  |  |
| 7. Age | -.065 | -.113 | **-.162*** | **-.255**** | .058 | **-.139*** |  |
| 8. Political views | .072 | .095 | **.230**** | .104 | -.043 | -.090 | -.010 |

Note. *p ≤ .05; **p < .01. Dummy variables coded: Risk/Uncertainty (1=Risk); Gender (1 = male); Income (1=below average)

Table S6. Pearson r correlations for the acceptable inflation rate and other variables, the occupational diseases condition (*N*=152)

|  | 1 | 2 | 3 | 4 | 5 | 6 | 7 |
| --- | --- | --- | --- | --- | --- | --- | --- |
| 1. Inflation |  |  |  |  |  |  |  |
| 2. Risk/uncertainty | -.123 |  |  |  |  |  |  |
| 3. Gender | .017 | .065 |  |  |  |  |  |
| 4. Income | -.086 | **.151*** | -.087 |  |  |  |  |
| 5. Fear about getting seriously sick | .079 | -.089 | **-.272**** | **-.137*** |  |  |  |
| 6. Personal control over getting sick | .065 | -.065 | .000 | .046 | .003 |  |  |
| 7. Age | -.046 | -.105 | -.171 | **-.246**** | .057 | **-.138*** |  |
| 8. Political views | .111 | .114 | **.211*** | .124 | -.033 | -.094 | -.010 |

Note. *p ≤ .05; **p < .01. Dummy variables coded: Risk/Uncertainty (1=Risk); Gender (1 = male); Income (1=below average)
